# Supplementary material for: Exploiting CRISPR-Cas to manipulate Enterococcus faecalis populations
Source: eLife. 2017 Jun 23;6:e26664. doi: 10.7554/eLife.26664 (PMC5491264; doi:10.7554/eLife.26664)
Supplement: Supplementary file 3. — DOI: http://dx.doi.org/10.7554/eLife.26664.023 [file elife-26664-supp3.docx]

| **Organism** | **Strain Name** | **Description** | **Ref** |
| --- | --- | --- | --- |
| ***E. coli*** | EC1000 ^a^ | *E. coli* cloning host, providing *repA in trans.*F- , *araD139 (ara ABC-leu)7679, galU, galK, lacX74, rspL, thi, repA* of pWV01 in *glgB, km* | (78) |
|  | JM109 | *E. coli* cloning host*, F´ traD36 proA+B+ lacIq Δ(lacZ)M15/ Δ(lac-proAB) glnV44 e14- gyrA96 recA1 relA1 endA1 thi hsdR17* | New England Biolabs |
| ***E. faecalis*** | V583 ^a^ | MDR bloodstream isolate, resistant to vancomycin, gentamicin, and erythromycin | (31) |
|  | V649 | V583 + *cas9/*tracrRNA | This study |
|  | V485 | V649 with the CRISPR2 terminal repeat replaced with a direct repeat | This study |
|  | V254 | V649 ΔP_CRISPR2_ | This study |
|  | Merz96 ^a^ | MDR bloodstream isolate, resistant to vancomycin, gentamicin, tetracycline, and erythromycin | (32) |
|  | M236 | Merz96 + *cas9/*tracrRNA | This study |
|  | ATCC 4200RF | Spontaneous Rifampicin and Fusidic Acid resistant derivative of ATCC 4200 | This study |
|  | ATCC 4200 Δ*cas9* | ATCC 4200RF with an in frame deletion of *cas9* | This study |
|  | OG1RF ^a^ | Oral isolate, resistant to rifampicin and fusidic acid | (29, 79) |
|  | CK111(pCF10-101) ^b^ | Spectinomycin resistant derivative of OG1 containing *repA in trans.*  Utilizes pCF10 ΔoriT to mobilize shuttle vectors containing pCF10 oriT | (27) |
|  | CK111SSp (pCF10-101) | Spontaneous Streptomycin resistant derivative of CK111(pCF10-101) | This study |
|  | DS5 | Isolate from unknown source, erythromycin resistant | (30) |
|  | C173 | CK111SSp(pCF10-101) with *ermB* disrupting the *cas9* locus. | This study |
|  | T11RFΔCR3cas9 | T11RF with an in-frame deletion of CRISPR3-*cas9* | (21) |
|  | T11CR1 | T11RFΔCR3*cas9*+CR1*cas9* | (21) |
|  |  |  |  |
|  |  |  |  |
|  | **Plasmid** | **Description** | **Ref** |
|  | pLT06 ^a^ | Carries temperature sensitive *repA* to select for chromosomal integrants and *pheS* to select for plasmid-free cells | (74) |
|  | pHA101 | pLT06 + oriT | (73) |
|  | pWH03 | pLT06 containing homologous fragments to GISE | (80) |
|  | pG19 | pWH03 + CRISPR1-*cas9*/tracrRNA | (21) |
|  | pVP31 | pLT06 derivaitve used to delete CRISPR1- *cas9* | This study |
|  | pMR23 | pHA101 derivative used to knock in the wild-type V583 CRISPR2 locus to its native genomic position | This study |
|  | pMR23 R-67-R-12-R | pHA101 derivative used to knock in the V583 CRISPR2 locus with the terminal repeat replaced with a direct repeat | This study |
|  | pMR28 | pHA101 derivative used to delete the promoter of the CRISPR2 transcript from the V583 chromosome | This study |
|  | pGE17 | Gibson-assembled plasmid used to insert *ermB* into the *cas9* locus of CK111SSp | This study |
|  | pMSP3535 ^a^ | Nisin inducible cloning vector for use in lactic acid bacteria | (75) |
|  | pAS201 | pMSP3535 + CRISPR1-*cas9* | This study |
|  | pGEM | Commercially available *E. coli* cloning vector | Promega |
|  | pLZ12 ^c^ | *E. coli/*Streptococcal shuttle vector | (26) |
|  | pKH12 | pLZ12 + oriT | This study |
|  | pKHSX | pKH12 with spacer X + NGG PAM | This study |
|  | pCR2 | pKH12 containing the V583 CRISPR2 locus | This study |
|  | pCR2-*ermB* | pCR2 with a spacer targeting *ermB* | This study |
|  | pCR2-Phage1 | pCR2 with a spacer targeting prohage Phage01 | This study |
|  | pAM771 ^a^ | non-cytolytic derivative of pAD1 mutagenized with Tn*917,* carries erythromycin resistance | (54) |

**Supplementary file 3. Strains and plasmids used in this study**

^a^ Strain or plasmid provided by Dr. Michael Gilmore of Massachusetts Eye and Ear Infirmary and Harvard Medical School.

^b^ Strain provided by Dr. Gary Dunny of University of Minnesota

^c^ Plasmid provided by Dr. Breck Duerkop of University of Colorado School of Medicine.
