# Supplementary material for: Exploiting CRISPR-Cas to manipulate Enterococcus faecalis populations
Source: eLife. 2017 Jun 23;6:e26664. doi: 10.7554/eLife.26664 (PMC5491264; doi:10.7554/eLife.26664)
Supplement: Supplementary file 4. — DOI: http://dx.doi.org/10.7554/eLife.26664.024 [file elife-26664-supp4.docx]

| **Primer Name** | **Sequence (5'-3')** | **Use** |
| --- | --- | --- |
| SalI OriT For | NNNNNNGTCGACCGCCAACGAATCGCC | Insert oriT to pKH12 |
| SalI OriT Rev | NNNNNNGTCGACGCAGCGTTTCTTTGAATAGG | Insert oriT to pKH12 |
| S4 Lin For | GAGATGCAGTTCTATGGTGACCATCTGTGCCAGTTCG | Create pKHS4 |
| S4 Lin Rev | TAAAGGCTCCGTATTCGTCTGACGACCAAGAGAGCC | Create pKHS5 |
| S11 Lin For | GATTTACGTTATATGGTGACCATCTGTGCCAGTTCG | Create pKHS11 |
| S11 Lin Rev | ACAGTCTTTTGTAAGTCGTCTGACGACCAAGAGAGCC | Create pKHS11 |
| S19 Lin For | GAACCGGTGGATGTGGTGACCATCTGTGCCAGTTCG | Create pKHS19 |
| S19 Lin Rev | CCGCAGTCCCCACTTTTGTCTGACGACCAAGAGAGCC | Create pKHS19 |
| S67 Lin For | GCATAATGACTATATGGTGACCATCTGTGCCAGTTCG | Create pKHS67 |
| S67 Lin Rev | TGAACAAGGGTTTGTTGTCTGACGACCAAGAGAGCC | Create pKHS67 |
| S96 Lin For | ACGCACTTACTTCTAGGTGACCATCTGTGCCAGTTCG | Create pKHS96 |
| S96 Lin Rev | TAAAAAACTACCTAAAGTCTGACGACCAAGAGAGCC | Create pKHS96 |
| S119 Lin For | CACATCAAGTTTCTGGTGACCATCTGTGCCAGTTCG | Create pKHS119 |
| S119 Lin Rev | CTACACCTTTAGCCCCAGTCTGACGACCAAGAGAGCC | Create pKHS119 |
| S244 Lin For | TGCAGCTGCAGCCTGGTGACCATCTGTGCCAGTTCG | Create pKHS244 |
| S244 Lin Rev | TATCTGCAAGACCCGCAGTCTGACGACCAAGAGAGCC | Create pKHS244 |
| SL Lin For | GCTTTTTCTCCCCTTGGTGACCATCTGTGCCAGTTCG | Create pKHSL |
| SL Lin Rev | CAAATCAAAAAAGTTTGTCTGACGACCAAGAGAGCC | Create pKHSL |
| NotI Cas9 For | NNNNNNGCGGCCGCGCAAACACAGTTAACCACG | Insert *cas9*/tracrRNA into pWH03 (pG19) |
| NotI Cas9 Rev | NNNNNNGCGGCCGCCTAGTCGACAACCTTTCTGC | Insert *cas9*/tracrRNA into pWH03 (pG19) |
| pGE17 Erm For | CTTTAGCTCCTTGGAAGC | Gibson Assemble pGE17 |
| pGE17 Arm2 For | ATACTACTGACAGCTTCCAAGGAGCTAAAGTTGTCGAAGAAATTAATCAATGGTATCTACG | Gibson Assemble pGE17 |
| pGE17 Arm2 Rev | GTATGTTGTGTGGAAACGTGGTTGCGACCACAC | Gibson Assemble pGE17 |
| pGE17 ori For | TGGTCGCAACCACGTTTCCACACAACATACGAG | Gibson Assemble pGE17 |
| pGE17 ori Rev | CTGGGTTTATCGACCCTGTCAGACCAAGTTTACTC | Gibson Assemble pGE17 |
| pGE17 pheS+Cat For | AACTTGGTCTGACAGGGTCGATAAACCCAGCGAAC | Gibson Assemble pGE17 |
| pGE17 pheS+Cat Rev | GAATTTTTTTAAATCTTCCAAGTTAAGGGATGCAGTTTAAAAATG | Gibson Assemble pGE17 |
| pGE17 Arm1 For | GCATCCCTTAACTTGGAAGATTTAAAAAAATTCAAACGATTTATTCG | Gibson Assemble pGE17 |
| pGE17 Arm1 Rev | AAGATACTGCACTATCAACACACTCTTAAGTTCCCCAGCCAGTGTAGT | Gibson Assemble pGE17 |
| pGE17 Erm Rev | CTTAAGAGTGTGTTGATAGTG | Gibson Assemble pGE27 |
| 12 Lin Rev | AGGGGAGAAAAAGCCAAATC | Linearize pMR23 to replace DR with TR |
| CR2 Down Lin For | CTCAAGCAATCCGTAATTTTC | Linearize pMR23 to replace DR with TR |
| 4200 Delcas9 Arm1 Rev | NNNNNNCTGCAGCTTCATTTTATTTCCACTCCTT | Create plasmid to delete CR1-cas9 (pVP31) |
| 4200 Delcas9 Arm1 For | NNNNNNCCCGGGGCACCAAGCCAACGAATTTA | Create plasmid to delete CR1-cas9 (pVP31) |
| 4200 Delcas9 Arm2 For | NNNNNNCTGCAGGTTGTCGACTAGATGGGCTG | Create plasmid to delete CR1-cas9 (pVP31) |
| 4200 Delcas9 Arm2 Rev | NNNNNNTCTAGAGCGAGCCAACATTGCTTTATTA | Create plasmid to delete CR1-cas9 (pVP31) |
| 4200 cas9 compF | NNNNNNCTGCAGGGCAAACACAGTTAACCACG | Create plasmid to compliment *cas9* deletion (pAS201) |
| 4200 cas9 compR | NNNNNNCTCGAGGCAAACGCTTATCATCGCAA | Create plasmid to compliment *cas9* deletion (pAS201) |
| PCKO ARM1 FOR | NNNNNNGAATTCAAAGAGAGGGACACGGATGC | Create plasmid for CRISPR2 promoter deletion (pMR28) |
| PCKO ARM1 REV | NNNNNNGCATGCGGCGGAAAAACCCACCATTT | Create plasmid for CRISPR2 promoter deletion (pMR28) |
| PCKO ARM2 FOR | NNNNNNGCATGCGCTATGGATAAGTGATGCGA | Create plasmid for CRISPR2 promoter deletion (pMR28) |
| PCKO ARM2 REV | NNNNNNGAATTCTCCCAAGTAAAGATCCACAAACCT | Create plasmid for CRISPR2 promoter deletion (pMR28) |
| CR2KI For | NNNNNNGGATCCTTCATTTGCAAGTCGCCAGC | Create plasmid for CRISPR2 knockin (pMR23) |
| CR2KI Rev | NNNNNNGGATCCAAAAAATAATTCTCCGAG | Create plasmid for CRISPR2 knockin (pMR23) |
| CRISRP2 Seq For | CTGGCTCGCTGTTACAGCT | Routine amplification of CRISPR2 |
| CRISPR2 Rev (com3 For) | ACTTATCACTTGATTAGTTTTCG | Routine amplification of CRISPR2 |
| CRISPR2 Up Lin Rev | CTCGGAGAATTATTTTTTCTCCATG | Linearize pCR2 to insert repeat-spacer-repeat unit |
| CRISPR2 Down Lin For | CTCAAGCAATCCGTAATTTTC | Linearize pCR2 to insert repeat-spacer-repeat unit |
| qrecA For | TGGTGAGATGGGAGCGAGCC | qPCR for *recA* |
| qrecA rev | TCAGGATTTCCGAACATCACGCC | qPCR for *recA* |
| qclpX For | AGGTAAAACTTTCTTGGCTCAAACG | qPCR for *clpX* |
| qclpX Rev | TTTCAACATCTTCCCCTACATAACC | qPCR for *clpX* |
| Primer extension | ACAGGGGAGAAAAAGCCAAAT | Primer for primer extension |

**Supplementary file 4. Primers used in this study**. Restriction sites are underlined
